# COPD management in primary care: is an educational plan for GPs useful?

**DOI:** 10.1186/2049-6958-8-24

**Published:** 2013-03-19

**Authors:** Enrica Bertella, Alessandro Zadra, Michele Vitacca

**Affiliations:** 1 Respiratory Rehabilitative Division Fondazione Salvatore Maugeri IRCCS, Via Giuseppe Mazzini, 129, Lumezzane, BS, 25066, Italy

**Keywords:** Continuity of care, Educational, General practitioner, Management

## Abstract

**Background:**

GPs currently deal with COPD*.* The aim of this study was to review COPD management, data collection in medical records, and adherence to GOLD guidelines of 12 GPs from rural areas of Northern Italy and to assess changes after an educational program (EP).

**Methods:**

From 2004 to 2008 medical records of patients, defined as COPD by GPs, were analyzed. Data collection in terms of tests prescription, Forced Expiratory Volume at first second (FEV_1_), smoking habits and actual drug treatment were reviewed at baseline and 1 year after EP.

**Results:**

437 patients were defined as COPD. GPs prescribed more chest X-rays than spirometry (99% vs. 74%, p<0.001), FEV_1_ was registered only in 50% of the population. GPs prescribed “correct” or “doubtful” (not related to FEV_1_) therapy in 38% and 56% of patients, respectively. Only smoking habit registration increased significantly (p<0.05) after EP.

**Conclusions:**

Adherence to COPD Guidelines is suboptimal and data collection is poor. The EP did not change significantly GPs’ practice: i) COPD diagnosis is largely clinical, ii) usage of spirometry is poor, GPs prescribe more chest X-rays iii) a small proportion of patients receive respiratory therapy, iv) therapy is often incorrect or not related to FEV_1_, v) correct clinical practice is influenced by the number of COPD patients and number of dedicated visits.

## Background

The prevalence of Chronic Obstructive Pulmonary Disease (COPD) is increasing worldwide [1]. In Italy, the majority of patients affected by COPD or presenting symptoms of obstructive airways disease refer to General Practitioners (GPs) for treatment. There are concerns of under-diagnosis and under-treatment of COPD in primary care setting. Symptoms indicating COPD should be interpreted properly by GPs and a definitive diagnosis should be confirmed by pulmonary function tests according to recommended guidelines [2-4]. Under-diagnosis has been reported due to under-presentation of symptoms by patients or lack of diagnosis by physician but also over-diagnosis might occur [2,5,6]_._

In previous studies, it has been reported that patients with suspicion of COPD never performed a spirometry nor visited a respiratory specialist [2,6].

GPs examine a large number of patients with different and multiple conditions and in this complex setting they can have difficulties in adhering to every disease-specific guidelines [7].

People with unrecognized COPD may miss the opportunity to improve their health status because of lack of prescription of functional tests [8].

It has been reported that GPs continue to be unaware of COPD guidelines and other information related to COPD, suggesting the need for continuing educational programs [8].

A recent paper [9] has showed that in 139 GPs following 454 patients, guideline adherence does not seem to impact symptom prevalence, exacerbation rate or lung function decline after one year of follow up.

Furthermore, a study conducted on 617,280 patients registered involving 400 Italian GPs, revealed that treatment was usually prescribed without performing pulmonary function tests and/or without taking into account the severity of airway obstruction [10]. More recently another Italian study [11] showed a poor relationship between the recommendations of the GOLD international guidelines and current clinical practice performed by pulmonologists.

When COPD has been diagnosed, a plan for clinical management is necessary because planned care can improve quality of life and reduce the economic burden related to hospital admissions and emergency visits [12].

To this end, we designed the current study with the aims of:

1. reviewing data collected by GPs in the medical records of their COPD patients

2. assessing the extent of under-treatment and adherence to GOLD guidelines for diagnostic and therapeutic procedures in a rural area

3. investigating whether management can be affected by an educational program.

## Methods

### Setting

The survey was carried out between 2004 and 2009 in the GPs’ office in a mountains/rural area with iron and steel industries of the Lombardy region (Val Trompia - Brescia) covering an area of 380 square kilometers [2], with 114,081 inhabitants.

In this area, a hospital for acute patients with emergency room is available and 70 GPs are employed in public home health services. Since 2006, a new specialized rehabilitative hospital (Fondazione Salvatore Maugeri, IRCCS for chronic diseases) has been offered to the territory.

Spirometry is easily available at both the above mentioned hospitals and other 2 pulmonary pathophysiology services at Spedali Civili in Brescia. Gp’s Prescription is required. The distance from any GP’s office to these hospitals is no more than 20 Km.

### Clinical data

Clinical information was retrieved by the Health Search Database (HSD) used by participating GPs. Since 2005, all GPs of this area had HSD, were well trained to use this standardized system and registered clinical data in the patient’s electronic files. The HSD contains information on patients’ demographics, medical records and drug history including records of preventive measures. All diagnoses were coded according to the 9^th^ revision of the International Classification of Disease (ICD-9) [13].

All data were extracted with a specific query from a dedicated software named Millewin: the query was sent to all GPs and the results were reversed in an Excel file to be analyzed.

Information on drugs was coded according to the latest version of Anatomical Therapeutic Chemical (ATC) classification system [14]. No data were available on symptoms.

The study was organized in two parts: 1. retrospective collection of basal data before an educational program (pre-education); 2. re-evaluation of clinical data 1-year after an educational plan on COPD knowledge and treatment. At the time of the study no specific COPD guideline dedicated to GPs was available.

### Data collection

#### Retrospective analysis (Pre-educational program)

The data analysis was conducted at Fondazione Salvatore Maugeri by a respiratory specialist.

All medical records of patients with diagnosis of COPD, as defined by GPs in the period 2004–2008, were retrospectively evaluated and reviewed at the end of October 2008.

The following data were considered for analysis: 1. spirometry and chest X–rays prescribed. 2. Forced Expiratory Volume at first second (FEV_1_) value and smoking habits registered in the previous 5 years (from 2004 to end 2008). 3. drug treatment regarding the last year of the abovementioned period. GPs’ prescription for drug treatment was evaluated in accordance with GOLD guidelines [1].

“Correct” prescription was defined as: a) No regular treatment at stage I b) Regular treatment with long-acting anti-muscarinic agents (LAMA) or long-acting bronchodilator agents (LABA) (or both) in symptomatic patients at stage II c) Regular treatment with inhaled corticosteroids (ICS) on top of long-acting bronchodilators, in general as fixed-dose combinations in patients at stages III and IV.

“Incorrect” prescription was defined as: a) regular treatment with LABA or LAMA alone at stages III, IV; b) only SABA at stage II and III; c) LABA or LAMA or ICS at stage I; d) ICS at stage II e) ICS on top of long-acting bronchodilators at stages I and II; f) no drugs were prescribed; g) theophylline was prescribed alone.

When GPs prescribed therapy was without registration of FEV_1_, the therapy was considered “doubtful”*.*

### Continuous educational program

The educational program was performed in January 2009. It was organized on 3 major points:

1) *face to face lectures on disease knowledge and treatment*: GPs attended two series of learning sessions lasting 3 hours performed by respiratory specialists from FSM. These sessions focused on COPD International guidelines [1]. Verbal information was accompanied by a written teaching manual adapted from the “Living Well with COPD” program [15].

2) *skills implementation strategy*: GPs visited our Hospital and attended the pathophysiology laboratory for a couple of hours to implement their knowledge, utility and modality of the spirometry test.

3) *second medical opinion:* respiratory specialists offered to GPs (by telephone) their clinical consultation when needed.

### Analysis of data after the educational program

In January 2010, one year after the educational program, the clinical parameters collected before the educational program, were re-evaluated.

### Statistical analysis

Data were reported as number, percentage or mean ± SD. Paired Student *t*-test and *χ*-square were used for comparison as appropriate. P-values< 0.05 was considered statistically significant.

## Results

12 GPs participated to the study, 2 were female, the mean age was 52 years (range 45–56).

### Data Collection and Retrospective Analysis (Pre-Educational Program)

At the end of 2008, medical records of 18,024 patients were reviewed and 437 patients were defined affected with COPD (2.42%) by GPs. The mean age was 69.8±12.9 years, 151 were females (35%).

Table 1 shows the basal data of COPD patients retrieved from HDS within a 5 years period (2004–2008). GPs prescribed more chest X-rays than spirometry (99% vs. 74%, p< 0.001) and FEV_1_ was registered only in 215 patients (49.2%). It was only in these patients that the severity of COPD, according to GOLD guidelines, could be classified. 91% of COPD patients were GOLD I or II. Smoking registration was available for 323 patients (73.9%). Only 188 out of 437 (43%) patients, defined as COPD, received some respiratory therapy. Differences in data collection were observed among GPs: spirometry has been performed from 14% to 76% of COPD patients and FEV_1_ has been registered from 0% to 76%.

**Table 1:** Clinical data of the patients’ population (437) registered in the GPs’ HDS database

	**Pre-educational program**
	**2004-2008**
***CHEST XRs***	
# of pts (%)	433 (99)
# of exams/pt/year±SD	0.5 ± 0.6
***SPIROMETRY***	
# of pts (%)	325 (74.3)°
# of exams/pt/year±SD	0.3 ± 0.4
***FEV1 registered***	
# of pts (%)	215 (49.2)
% of patients/all pts with spirometry	66.1
***FEV1 % prd***	81.0 ± 21.0
***GOLD I %***	43
***GOLD II %***	48
***GOLD III %***	8
***GOLD IV %***	1
***SMOKING REGISTRATION***	
# of pts (%)	323 (73.9)
# current smokers (%)	32 (7.4)
***VISITS TO GP*** per pt, per year	
Mean±SD	17.9 ± 13.4

*Legend:* P = 0.000 Spirometry vs Chest XRs.

### Continuous educational program

All 12 GPs attended the educational sessions and Hospital visits. During 1-year activity, 7 out of 12 GPs required a second opinion performing 12 calls to the respiratory specialists.

### Analysis of data after educational program

109 patients (25%) were lost to follow- up because of death, change of GPs or residence; baseline data of these patients did not differ from those still in the study.

328 patients (75% of the baseline group) remained in the study. The mean age was 69 ± 12.2 years, 107 were female.

Table 2 shows clinical data registered by GPs in the 328 patients present in the HDS before and after the educational program.

**Table 2:** Clinical data registered in the GP’s HDS database for 328 patients available before and after the educational program

	**2008**	**2009**
***CHEST XRs***		
# of pts (%)	101 (31)	93 (28)
# of exams/pt±SD	0.44 ± 0.82	0.37 ± 0.69
***SPIROMETRY***		
# of pts (%)	64 (19)°	55 (17)°
# of exams/pt±SD	0.23 ± 0.53	0.19 ± 0.46
***FEV1 registered***		
# of pts	49	47
% of patients/all 328 pts	15	14
% of patients/all pts with spirometry	76.6	85.5
***FEV1 % prd***	77.0 ± 38.0	79.0 ± 26.0
***GOLD I %***	41	45
***GOLD II %***	49	38
***GOLD III %***	8.2	15
***GOLD IV %***	2	2
***SMOKING REGISTRATION***		
# of pts (%)	255 (78)	298 (91)*
current smokers (%)	34.1	32.5
***VISITS TO GP*** per pt, per year (mean±SD)	17.9 ± 12.1	9.15 ± 13.2

* p = 0.000 Educational program vs pre-educational program; and ° p =0.000.

Spirometry vs Chest XRs.

The number of Chest X-rays performed was significantly higher than that of spirometry (p< 0.001) at both time points. After the educational program, prescriptions of Chest X-rays and spirometry did not change significantly. 39 out of 55 patients (71%) who performed spirometry during 2009 had not performed it in the previous year and only 15 out of 55 patients (27%) had never performed it either in the previous year or in the last five years. In 33% of spirometry, FEV_1_ was not registered. Smoking habits registration increased significantly from 78 to 91% (p< 0.05). Also after the EP, differences were observed among GPs. Only 8 GPs improved spirometry prescription (improvement was defined both for the total number of prescriptions and the presence of at least 1 new prescription) and 5 improved FEV_1_ registration (from 2%to 20%): a correlation was found between the number of patients followed by the GP and the number of FEV1 registered (p = 0.023).

Table 3 shows drugs prescription data in 328 patients present in the HDS before and after the educational program.

**Table 3:** Data related to actual drug treatment for 328 patients available before and after the educational program

	**2008**	**2009**
# of pts on regular treatment (%)	127 (38.7)	109 (33.2)
# of pts on *correct* therapy (%)	48 (14.6)	48 (14.6)
% of patients/pts receiving therapy	37.8	44
# of pts on *incorrect* therapy	8 (2.4)	9 (2.7)
% of patients/pts receiving therapy	6.3	8.2
# of pts on *doubtful* treatment	71 (21.6)	52 (15.8)
% of patients/pts receiving therapy	55.9	47.4
# of pts on oxygen therapy	2	2

Correct, according to GOLD guidelines; incorrect=, irrespective of GOLD guidelines; doubtful=, not related to FEV_1_ severity registration.

In 2008, only one third of these patients received respiratory therapy. GPs prescribed “correct”, “incorrect” or “doubtful” therapy in 38%, 6% and 56% of cases respectively. 75 patients (23% of the whole population) had the FEV_1_value reported, but received no therapy despite the evidence of airway obstruction. No significant changes in the therapeutic approaches were observed after the educational program.

The most frequent mistake was the overuse of steroids and theophylline in mild obstruction and the prescription of only short acting β_2_ agonists in GOLD II and III.

**Figure 1:**
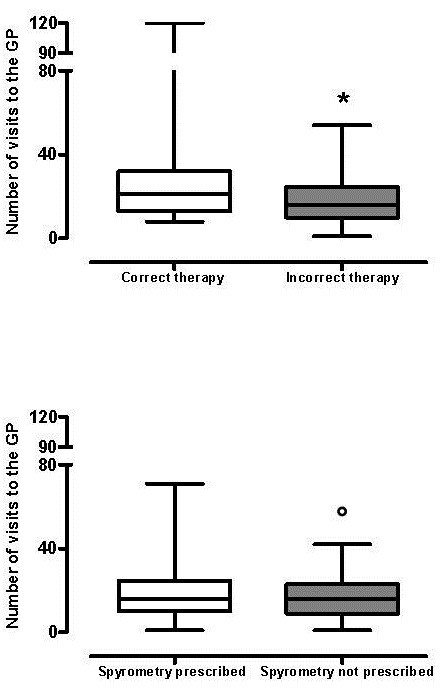
**Number of visits to GPs in patients with correct or incorrect therapy at the end of 2008 (TOP) and in patients with or without spirometry prescription (BOTTOM) during 2004–2008.** * p=0.025 °p=0.029.

Patients receiving the correct therapy in 2008 (Figure 1, Top panel left) and patients who performed at least one spirometry during the previous five years (Figure 1, Bottom panel left), underwent a significantly higher number of GPs examinations compared to patients with incorrect therapy (Figure 1, Top panel right, p=0.025) and without spirometry (p<0.03) (Figure 1 Bottom panel right, p=0.029). Similar data were found after the educational program.

## Discussion

This study shows that adherence to the international COPD Guidelines by Italian GPs is suboptimal and data collection in medical records is poor and incomplete. Moreover, the educational program had a very limited impact on GPs’ clinical practice. Indeed, in primary care setting: i) the diagnosis of COPD is still largely clinical, ii) the usage of spirometry is poor with GP tending to perform more chest X-rays than pulmonary function tests, iii) a small proportion of patients receive respiratory therapy, iv) the prescribed therapy is often incorrect or not related to FEV_1_, v) correct clinical practice is influenced by the number of COPD patients followed and the number of dedicated visits.

In this study the prevalence of COPD in this area was around 2.4%, which is a little less than that estimated in the general population (5%) [13,14,16] but similar to the Italian data reported by Cazzola *et al. [*[10]. The under-diagnosis of COPD in primary care medicine is probable and has already been described in literature [2]. Despite the educational sessions, in 2009 the number of patients who performed a spirometry was similar to that in 2008, 55 (17%) vs. 64 (19%), respectively, and only 15 patients performed a spirometry for the first time in 2009. The reason of this lack of improvement in spirometry testing is not clear. It could be due to the workload of the GPs or to the necessity of cost saving. On the other hand, spirometry could be perceived by GPs as not useful for determining starting therapy, since treatment is based on symptoms. The latter explanation is supported by the relation found between the number of visits to the GPs and the prescription of spirometry and “correct” therapy: higher the number of GP contacts, higher the probability for the patient to be submitted to spirometry and correct therapy.

The number of patients who had FEV_1_ value registered was even lower before the educational sessions. After these latter ones, the number of registered FEV_1_ in patients with a spirometry increased. So, despite a poor improvement in the prescription of pulmonary function tests, for GPs the importance to classify severity of COPD was clear. It is noteworthy that the patients with FEV1 registered had mostly mild to moderate disease, thus it is possible that GPs refer more severe patients to specialists without reporting the results of tests and visits. This could explain the large number of patients under therapy without registration of FEV1.

On the other hand we found that GPs with higher number of COPD patients tend to register FEV_1_ more frequently. This brings out the importance of experience and correct case funding in the management of a particular disease.

Smoking habits is a major risk factor for COPD: GPs are aware that smoking habits must be registered. However, in 22.3% of patients this data was not registered. Smoking registration increased significantly to approximately 90% after the educational sessions, but the number of current smokers (34.1%) decreased only slightly after the educational program (32.5%).

The data about treatment were surprising. Prior to the educational program the percentage of patients receiving therapy for COPD, either “correct” or “incorrect”, was very small (38.7%). This data would not have been underestimated because therapy is routinely prescribed by GPs even if under specialist’s advice. Of these patients under treatment, more than 50% received therapy probably not related to FEV_1_. It is possible that the GPs have looked at spirometry without reporting the data or that the therapy have been prescribed by a specialist and only reported by the GP. In any case, it is not possible to state if the therapy taken by these patients was “correct”. Among 56 patients who had registration of FEV_1_ and received therapy, 48 received drugs appropriate for the severity of obstruction and eight received an “incorrect” therapy. The fact that the most frequent mistake was the abuse of steroids, theophylline or short acting β_2_ agonists may be explained with the hypothesis that GPs often start therapy after a relapse and subsequently maintain the same therapy without further investigations. The large number of untreated COPD cases could possibly reflect incorrect diagnoses.

After the educational sessions, the number of patients receiving therapy decreased. In particular, the reduction was among the group of patients without FEV1. This made us believe that the reason could be a cessation of therapy by the GPs pending a definitive diagnosis. In any case, it is noteworthy that patients (less that 15%) with an appropriate therapy were very few indeed.

Table 2 shows that the mean number of visits/patient was 17.9 in 2008 and 9.15 in 2009. In our opinion this reduction could be a positive sign because patients felt better and had less exacerbations and did not need to visit the GP. This hypothesis would agree with a recent study [17] reporting that respiratory medications prescribed in COPD complied poorly with the GOLD pharmacologic treatment guidelines, but were correlated with the number of prior respiratory healthcare visits.

In the management of COPD, a discrepancy between prescribed therapy and guidelines has been observed in several previous studies [9-11,18,19].

Our results are similar to that observed both in Italy and worldwide [6,9-11]. A recent Italian study [11] conducted by pulmonologists showed a poor relationship between the recommendations of the GOLD international guidelines and current clinical practice: out of 44,094 patients recruited, a total of 302 (7.4%), of whom 263 at stages I and II and 39 at stages III and IV, did not receive any regular pharmacological treatment for COPD; the remaining 23,792 (92.6%) received at least 1 drug on a regular basis.

GPs seem to pay more attention to the treatment of symptoms than to the diagnosis of a chronic condition. Even though symptoms are important to suspect COPD and follow its evolution, they are not sufficient to make a diagnosis of COPD. GOLD guidelines [1] indicate spirometry as the only evaluation to confirm or exclude airflow obstruction, this being the most important diagnostic criteria of COPD. Moreover, post-bronchodilator FEV_1_ is needed to define the severity of the disease and thus to prescribe the “correct” therapy [1].

In a Swiss study [9] an inappropriate treatment for their stage of disease was found in 44% of all COPD patients after one year of follow up compared to 47% at baseline when treated by GPs. Different results were reported by a Danish study [19] which shows that substantial improvements can be achieved by focused education of GPs. A Spanish study conducted by Soler et al*.*[20] showed improvement in COPD diagnosis and management after a training program for GPs with an additional improvement when GPs were given a spirometric device. Other publications have supported the feasibility and use of spirometry testing by GPs [21,22].

### Implications for future research or clinical practice

We believe that more research must be done to improve COPD management in primary care. In addition, more structured and continuous educational program with practical training are necessary to address knowledge gaps and mistakes in daily practice. Technology such as personal electronic card and/or telemedicine can help to facilitate dialogue between GPs and specialists [23] and could offer new useful tools to improve the management of COPD by GPs as recently shown by Bonavia et al*.*[24]. GPs need support in COPD management; correct case funding and adequate numbers of dedicated visits are paramount to improve clinical practice.

### Limitations of the study

The main limitation of this study is that the standard for drug prescriptions was based on the GOLD guidelines [1]: these guidelines may not be universally accepted due to the emerging necessity to individualize drug therapy according to more sophisticated and holistic parameters rather than the pure FEV_1_ value.

A second limitation is the absence of symptoms registration in the database used, thus the only reference to evaluate treatment decisions was FEV_1_. However, a recent paper [9] has showed in 139 GPs that guideline adherence does not seem to impact symptom prevalence, exacerbation rate or lung function decline after one year of follow up.

Another important limitation is that this study is partly designed as retrospective. A retrospective analysis of a Health Search Database for GPs is limited by the fact that the physicians using the database were not trained to specifically register the variables needed for the present study. A negative result of the training course could be caused by under-registration of data by the physicians, in particular for possible visits performed by pulmonologists. On the other hand, this fact may be a strength of the study because GPs were not obliged to a specific protocol and the present data are the real life mirror of their habits.

## Conclusions

In conclusion, since the adherence to the COPD guidelines by Italian GPs is suboptimal and educational program on respiratory medicine had a very limited impact in the clinical practice, more integrated and synergistic hospital-GP strategies are recommended for improving management of chronic respiratory diseases.

## Abbreviations

ATC: Anatomical Therapeutic Chemical; COPD: Chronic Obstructive Pulmonary Disease; EP: Educational Program; FEV1: Forced Expiratory Volume at first second; GPs: General Practitioners; HSD: Health Search Database; ICD-9: International Classification of Disease; ICS: Inhaled Corticosteroids; LABA: Long-Acting Bronchodilators Agents; LAMA: Long-Acting Anti-Muscarinic Agents; SIMG: Italian College of General Practitioners.

## Competing interests

The Authors have no conflicts of interest to disclose.

## Body living ethics approval

Fondazione Salvatore Maugeri, IRCCS (Lumezzane, Brescia).
